# How research funding agencies support science integration into policy and practice: An international overview

**DOI:** 10.1186/1748-5908-9-28

**Published:** 2014-02-24

**Authors:** Pernelle A Smits, Jean-Louis Denis

**Affiliations:** 1 ENAP (École Nationale d’Administration Publique), 4750 Henri Julien, Montréal, Québec H2T 3E5, Canada; 2 Université de Montréal, C.P. 6128, succursale Centre-ville, Montréal, Québec H3C 3J7, Canada

**Keywords:** Knowledge management system, Knowledge funding agencies, Knowledge use/utilization, International, Science, Policy, Funding agency

## Abstract

**Background:**

Funding agencies constitute one essential pillar for policy makers, researchers and health service delivery institutions. Such agencies are increasingly providing support for science implementation. In this paper, we investigate health research funding agencies and how they support the integration of science into policy, and of science into practice, and vice versa.

**Methods:**

We selected six countries: Australia, The Netherlands, France, Canada, England and the United States. For 13 funding agencies, we compared their intentions to support, their actions related to science integration into policy and practice, and the reported benefits of this integration. We did a qualitative content analysis of the reports and information provided on the funding agencies’ websites.

**Results:**

Most funding agencies emphasized the importance of science integration into policy and practice in their strategic orientation, and stated how this integration was structured. Their funding activities were embedded in the push, pull, or linkage/exchange knowledge transfer model. However, few program funding efforts were based on all three models. The agencies reported more often on the benefits of integration on practice, rather than on policy. External programs that were funded largely covered science integration into policy and practice at the end of grant stage, while overlooking the initial stages. Finally, external funding actions were more prominent than internally initiated bridging activities and training activities on such integration.

**Conclusions:**

This paper contributes to research on science implementation because it goes beyond the two community model of researchers versus end users, to include funding agencies. Users of knowledge may be end users in health organizations like hospitals; civil servants assigned to decision making positions within funding agencies; civil servants outside of the Ministry of Health, such as the Ministry of the Environment; politicians deciding on health-related legislation; or even university researchers whose work builds on previous research. This heterogeneous sample of users may require different user-specific mechanisms for research initiation, development and dissemination. This paper builds the foundation for further discussion on science implementation from the perspective of funding agencies in the health field. In general, case studies can help in identifying best practices for evidence-informed decision making.

## Background

A key challenge facing health services research and science policy is understanding and optimizing the integration of science into both policy and the practices of professionals. This paper investigates how health research funding agencies support the integration of science into policy and practice.

First, there is confusion and misunderstanding about terms such as knowledge transfer, knowledge translation, implementation and research utilization, and even more so, about such concepts as ‘moving knowledge into action’ [1]. This has led to calls for an improved understanding of the uses of research [2]. Only a few fields of research—notably health service research [3-9] and knowledge transfer research [10,11] —have investigated science integration into policy and practice (SIPP). In fact, health services research, especially research concerned with the use and implementation of research results in policy, has become so intertwined with the field of knowledge transfer that calls for efforts have been made to compare and clarify terms used in the two fields.

Knowledge transfer spans a number of different fields, including communication with the public, communication with media, and collaborative approaches to science. In this paper, the main focus is the direct integration of science into decision making, both in policy and practice. We do not examine the indirect influences of science on policy making, such as lobbying activities and public opinion. Rather, we focus on evidence-informed decision making aimed at modifying policy and practice, such as SIPP and methods prior to SIPP in the process [12]. The line separating knowledge transfer and knowledge translation is unclear, with some authors advocating that concepts like technology transfer, continuing education and commercialization not be included under these terms [13]. We will not enter into this debate here; rather, we will focus on the importance of research-based knowledge in the decision-making process at the level of both policy and practice.

In the 1970s, concern over knowledge-based decision making increased among researchers. Research utilization [14] encompasses conceptual use, instrumental use and strategic purposeful use. Organizations—funding agencies in particular—have been interested in licensing, acquiring, and commercializing biomedical discoveries for years. Recently, stakeholders have championed the integration of health-related sciences, human sciences, and biomedical sciences into policy and practice. Dedicated funding, the identification of strategic objectives, and the implementation of internal activities within funding agencies all foster SIPP. In the literature on science policy, or how the policies of national governments, supranational organizations, public science funding organizations, and large public research organizations aimed at influencing the production of scientific knowledge [15],’ funding agencies have been analyzed [12,16,17].

How science policies for SIPP are ingrained in the research agenda at the national level [18-20], how they are adjusted to the educational sphere [21], how they are formatted under external pressures [22], and how they are included in citizen and democratic procedures are all important issues. The dialogue around, and actions aimed at, producing stronger investigative SIPP research highlights two elements: the need to consider other key players beyond just scientists and policy makers, and the benefits of understanding the influence of scrutinizing institutions on the role of science in policy making [23].

This paper identifies funding agencies as a key player and contributor to SIPP. This premise means we need to study funding agencies more closely. Not only do we need to see them as a key player, as already suggested by Sutherland [23], we need to study their role as a central element, rather than just one variable in the overall dynamics of SIPP.

This paper is innovative in several ways: it focuses on funding agencies as a key player in SIPP rather than just a component of the outside environment as previous authors have done [12,16,17]; it compares funding agencies on SIPP and examines the policy and practice achievements of program funding; it expands the traditional conceptualization that knowledge transfer is centered around practitioners, policy makers, and academics; it adds to the growing trend for research focused on policy integration in the broader framework of health service research; and it focuses on ‘the external social structures and processes that influence individual knowing and the production of collective knowledge’ [24].

In Western countries, the agendas of funding agencies prioritize national research and orchestrate research funds to answer current and emerging health challenges that have the potential to improve the health of their populations. Funding agencies constitute operational pillars for policy makers, researchers, and service delivery institutions like hospitals and health departments. This paper investigates the role of funding agencies in SIPP.

We provide an overview of the knowledge translation mechanisms of funding agencies as it relates to the health sector. There is a need to identify and discuss the challenges to integrating research into policy and into practice in a timely manner, and to enhance the integration of research findings into policy—at the macro, local and in practices at the micro levels.

To date, a few empirical studies have provided details about SIPP funding: most notably, a study that compared European health services research at the national level [5], and another that compared health research funding at the agency level [25]. Both studies relied on a small number of informants. In the first study, 34 countries were surveyed in 2007 to 2011, while in the second, members from 33 agencies were interviewed in 2003 and 2004. Both studies presented results from an in-depth analysis of official funding agency documents. The European comparisons mainly looked at both public and private funds, policy making, and the activities being promoted. In our study, we examine these aspects, but also add the documented benefits of funding, both in and outside of the policy domain. We include ‘internal’ SIPP orientation strategies by funding agencies, along with agency activities that support the innovation cycle for beneficiaries of funds. We present a comparative, international analysis of the funding agencies’ requirements, funding strategies, and the types of activities they fund. Therefore, this paper continues the international focus, while also examining the role that funding agencies play in SIPP. It also contributes to the literature on SIPP by emphasizing health research funding agencies and inward strategic positioning, all while serving to bring the current literature up to date.

The overall objective of this paper is to determine what are the intentions, actions, and benefits of the integration of science into policy and practice in health-related funding agencies throughout the world. Our comparisons will help answer questions like: What kind of visibility do funding agencies give to SIPP? How do funding agencies promote SIPP? What impacts do funding agencies have on practice and policy?

## Methods

### Selection of funding agencies

We selected funding agencies from six countries: Australia, The Netherlands, France, Canada, England, and the Unites States. The agency had to be national or supranational in scope, fund health-related research, and a major provider of research funds. We questioned professors, researchers, members of funding agencies, managers and health service providers who were mobilized to identify the characteristics of the major provider of research funds category, identifying the ‘contrast or continuum on knowledge translation engagement’ [25].

Granting councils are:

Australia

National Health and Medical Research Council (NHMRC)

Australian Research Council (ARC)

The Netherlands

The Netherlands Organization for Health Research and Development (ZonMW)

France

Institut National de la Santé et de la Recherche Médicale (INSERM)

Centre National de la Recherche Scientifique (CNRS)

Agence Nationale de la Recherche (ANR)

Canada

Canadian Institutes of Health Research (CIHR)

Natural Sciences and Engineering Research Council (NSERC)

Social Sciences and Humanities Research Council of Canada (SSHRC)

England

National Institute for Health Research (NIHR)

USA

Food and Drug Administration (FDA)

Environmental Protection Agency (EPA)

National Institutes of Health (NIH)

### Data sources

We selected published reports and information provided on the websites of the selected funding agencies (see Additional file 1). We extracted information from strategic plans, mission statements, organizational charts, reports on available funding for research and internal productions, success reports, and descriptions of agency activities. Given that the interest in SIPP is fairly recent, we restricted our search of key resources to the period of 2005 to 2011.

The empirical secondary data that we gathered from these sources was limited: these data do not necessarily reflect the most recent and emerging trends in the policies and decisions being made by funding agencies. To make up for this limitation, we searched for the latest available and accessible strategic plans and organizational documents. Such an approach may have introduced a positive bias and lead to a more selective discourse disproportionately centered around ongoing activities and the results obtained for these particular activities. However, we believe that this bias is less serious because we are interested in achievements rather than failures and barriers. We included reports on the impacts of funding on policy or practices when the data were detailed and relevant to the grant received. While source data analysis may not have allowed us to compile information on all of the internal dynamics of funding, such dynamics were not our main point of interest.

Using secondary data has the advantages of providing a level of depth similar from agency to agency, and sometimes these secondary data have similar categories, thereby making the comparisons more accurate and straightforward. The reports and information published on websites are produced after a review process and approval by the department responsible for editing and publishing official data, thereby ensuring a consistent, shared view on topics of interest and, ultimately a comprehensive data set. It would have been difficult to obtain such a large and diverse dataset any other way: a single representative from one institution simply could not have provided such variety in content. We believe reports and official website information are a useful dataset for studying SIPP in funding agencies at this point in the development of research about science integration into policy and health.

Note that our data collection relied on information available on the agencies’ websites. The uniformity of the data published by a particular agency cannot be guaranteed by our methodology. There is reason to believe the selected agencies publish data that are not so different from one another. Indeed, the websites reveal each agency’s formal representation of its own activities. Moreover, the information published by agencies on their websites is directed at the public, researchers, policy makers, and practitioners. It is their public portrait.

Our analysis of the websites generated secondary, tertiary, and endless snowball references as a result of navigating the agencies’ websites plus those of linked agencies and ministries. We pulled documents that were directly posted on the agencies’ own websites. Relevant information was either easily accessible via the homepage, or it was less available, being stored in archives or identifiable by the title on webpages. We browsed the whole website, including archives, paying particular attention to webpages related to strategy and funding. Of course, a key informant in each agency could have validated the documents we retrieved, but we sought only to examine documentation.

### Identification of variables

We investigated three main variables of SIPP and their corresponding dimensions (Table 1):

1. Intentions: Health research funding agencies set health as a priority and then devise ways to support and foster the integration of science into policy and practice. We examined the following dimensions of intentions: organizational visibility of SIPP (absent, external, internal) and the purpose of SIPP (management, network, capacity building).

2. Actions: Health research funding agencies organize both internal and external SIPP-related activities. Actions are any specific activities by funding agencies to ensure the materialization of science into policy and practice, and to encourage evidence-based or evidence-informed policy or practices. We examined the following dimensions of actions: type of external actions (push, pull, linkage and exchange (L&E) funding), types of internal actions (reinforcing capability, process, codification), and steps in the innovation process (synthesis, initiation, development, end of grant, science, training).

3. Benefits: Funding agencies collect evidence of the empirical benefits and return on investment for their funding activities. Benefits are the consequences—often positive outputs and outcomes—that contribute to, or are generated by, specifically targeted SIPP activities. We examined the following dimensions of benefits: type of influences (policy, practice, service), specific influences on policy (local, national, international), specific influences on practice (improvement, new, modification of regulations), and specific influences on service delivery (financial, human, material).

**Table 1:** Summary of variables

**Variable**	**Dimensions**
Intention	Organizational visibility
	Purpose of SIPP
Action	Type of external actions
	Type of internal actions
	Steps of innovation
Benefits	Type of influences
	Specific influences on policy
	Specific influences on practice
	Specific influences on service delivery

Policy refers to orientations taken by funding agencies on knowledge transfer. Practice refers to the operational translation of programs and activities.

## Results

The agencies used a variety of terms and concepts when talking about SIPP in their strategic and mission statements: testing knowledge, applying knowledge (application), transfer, translate research/knowledge, valorization, knowledge management, put to real-world use, integrate science and technology, science coordination with priorities, informed policy advice, use of knowledge/utilization of science, strategic management of licenses, innovation, mobilizing knowledge, commercialization, evidence-based approach, and consider results in the decision making process.

### Analysis of intentions

#### Organizational visibility

The importance and readiness of SIPP for funding agencies is partially reflected in their organizational charts, mission statements, and strategic objectives. The organizational charts sometimes attribute the responsibility of SIPP to distinct directorates, departments and/or branches (Table 2). For example, SSHRC has a separate division for knowledge mobilization and program integration, unlike ARC, which does not have any specific divisions. Most of the funding agencies manage their SIPP mission from within; usually it is under the responsibility of one of their directorates or branches. For example, SSHRC has a knowledge mobilization and program integration division, while NHMRC has research investment direction a /Knowledge Translation & Capacity Building. In France, INSERM manages some of its SIPP objectives through a private entity: INSERM Transfer SA. This group is in charge of putting highly promising biomedical innovations sponsored by INSERM funds into action; it diagnoses, accompanies, helps manage, protects, and supports innovations on the way to commercialization. One could say that INSERM is involved in translational research rather than knowledge transfer *per se*, although there needs to be some adoption of knowledge with partner companies and so this activity qualifies as knowledge transfer in our analysis.

**Table 2:** Organizational visibility of SIPP in funding agencies

**Visibility**	**Funding agencies**
Absent	ARC. no specific directions
External	INSERM. INSERM Transfert SA a private entity from INSERM
Internal	CNRS Direction of innovation and relationships with companies (translation)
	ANR. Partnership and competitiveness; investment in the future (translation)
	EPA Office of science policy
	NIH Office of science policy analysis
	CIHR Knowledge translation branch; partnerships and citizen engagement branch; communications and public outreach branch
	SSHRC Knowledge mobilization and program integration division
	NHMRC Research investment direction/Prog Grants, Knowledge Translation & Capacity Building
	NSERC Research partnerships program directorate; research grants and scholarship directorate

For ZonMw and NIHR, we found no data on organizational structure for SIPP. Please note that NIHR does commission research on SIPP through the NHS and the Health Services and Delivery Research Programme [35].

#### Purpose of SIPP

Strategic support for SIPP was embedded in various strategies devoted to management, exchange, and/or capacity building. SIPP could be narrowed down to management objectives, in which knowledge informs the development or improvement of new practices and getting a product out on the market. In such cases, the funding agencies put an operational emphasis on SIPP. For example, INSERM placed an emphasis on managing the transfer of technology:

‘Its principal mission is to coordinate the valorization of medical innovations from research laboratories’. [our translation]

Organizational chart: INSERM Transfert SA a private entity from INSERM

Source: translation. INSERM Transfert mission. Website.

Some of the funding agencies had a broader scope and emphasized certain processes involved in SIPP. They favored a flow of information through contacts and exchanges between researchers and policy makers. For example, CIHR referred to knowledge translation and insisted on the importance of dynamic and iterative exchanges between the two communities:

‘Knowledge translation (KT) is defined as a dynamic and iterative process […] to improve the health of Canadians, provide more effective health services and products and strengthen the health care system. This process takes place within a complex system of interactions between researchers and knowledge users which may vary in intensity, complexity and level of engagement depending on the nature of the research and the findings, as well as the needs of the particular knowledge user’.

Organizational chart: Knowledge translation branch; partnerships and citizen engagement branch; communications and public outreach branch

Source: CIHR. DPR 2009–2010.

SIPP may build on the influence it has on policy and policy makers to increase its influential capacity in the legislative branch. In Australia, both funding agencies we studied sought to contribute to individual and organizational learning: NHMRC by translating knowledge to a broad audience in order to improve policy and practice, and ARC by playing an advisory role in policy making.

‘Policy: To provide informed high quality policy advice to Government—through participation in policy forums and Government reviews, informed stakeholder consultations, evaluation and ongoing monitoring of performance’.

Organizational chart: No specific directions.

Source: ARC. 2010. Strategic Plan 2010–11 to 2012–13.

To summarize, our comparison of the intentions of funding agencies shows that:

1. Most funding agencies emphasized SIPP through specific organizational structures;

2. Science was integrated into policy and practice through various operational means and by direct support: through improvements to the management of SIPP, encouraging the flow of information between actors in SIPP, and improving learning related to knowledge;

3. The internal structure of the funding agencies, combined with relevant funds, increased the likelihood of SIPP.

### Analysis of actions

#### Types of external actions (pull, push and L&E models)

Informed decision making was often conceptualized in terms of how to encourage mechanisms that would bring science into the decision-making arena—that is in a push fashion. The push-pull model of knowledge translation is famous for distinguishing between mechanisms driven by science (push) and those driven by the demands of practitioners or policy makers (pull). These models consider two communities in interaction: the scientific community of researchers and the practice community of decision-makers, politicians, local community members, *et al*. A third model advocates for consideration of interactions that take place along the innovation cycle and make science useful [26]. Such L&E [10], or deliberate models, rely on the co-construction of applied knowledge [27] and the relevance of applied research to both users and researchers. During research development, data collection and interpretation tasks can be shared. Most of the agencies we examined funded programs based on one or two of these models, either push, pull, or L&E. Few provided funding based on all three types of SIPP models, with the exception of NHMRC, ZonMW, NSERC and NIHR (Table 3, line 1).

**Table 3:** Funding agencies program funds

**Line 1. Program funds in a push/pull/linkage and exchange (L&E) model**										
			**Push**		**Pull**		**L&E**		**3 modes**	
% of funding agencies			69		62		62		23	
**Line 2. Eligible expenses per innovation cycle steps**										
		**Synthesis**	**Initiation**	**Development**		**End of grant**		**Science**	**Training**	**All steps**
% of funding agencies		38	54	69		77		31	62	23
**Line 3. Internal activities related to SIPP**										
		**Capabilities**		**Process**		**Codification**			**All 3**	
% of funding agencies		85		54		46			46	
**Line 4. Benefits of funded projects**										
	**Policy**			**Practice**			**Service delivery**			**3 types**
	**Local**	**National**	**International**	**Improvement of practices**	**New**	**Modification of practice regulations**	**Financial**	**Human**	**Material**	**Policy, practice and service delivery**
% of funding agencies	15	8	8	31	31	8	8	8	23	8
	15			46			23			8

ZonMW’s Ethnic Minorities and Health Care Program belongs to the push model driven by science:

‘The Ethnic Minorities and Health Care Programme aims to promote the implementation of knowledge and skills available in this area. It targets both providers and users of care. The programme focuses on improving somatic curative care, an area in which knowledge and methods are available that could potentially improve care for ethnic minorities in the Netherlands. The aim is to spread this knowledge and these methods, and ensure they become part of mainstream care’.

NHMRC’s urgent research program reflects a pull model driven by policy needs:

‘Research that must be undertaken rapidly in response to a threat to public health. The threat may be generalised, or specific to a particular group of individuals, and may be identified as either a current major problem, a potential major problem or a problem that is expected to increase in the future. The main catalysts for urgent research will be the fact that a disease or illness, or its variant(s), is previously unknown or unidentified, and has a high morbidity and/or mortality rate, thus garners media coverage and public and/or governmental concern. This definition covers the range of possibilities at the cellular level (the identification of the disease or illness, and its variants), to those at the public and population level, and then the level of risk to a nation. For example, if a disease poses a probable or actual threat to the national and/or local economy by hindering exports, tourism, agriculture, and so on. The SRDC has outlined a process for considering requests for urgent research based on this decision’.

ANR’s competitive pole funding program belongs to the linkage and exchange model:

‘A competitive pole is constituted by companies, research laboratories and training centers in a geographic area in order to favor cooperation and exchange’. [our translation]

In the agencies studied, the creation and development of research in collaboration with users (L&E model) took the form of meetings to explore prospective subjects of interest, on-site training opportunities for practitioners to come to research settings, and inviting researchers to join decision making settings. The NIHR in England provided financial support for partnerships with health service providers through its Service Delivery and Organization (SDO), where research results could be presented to practitioners and practitioners could present their needs. AHSC Academic Health Science Centers have brought together research units, health care services, and education institutions, thus promoting cross-fertilization between research and practice.

Funding based on the push model was for the transfer of existing research to users via publications in peer-reviewed journals or practitioner-oriented journals, or through face-to-face dissemination in forums and conferences. For example, the EPA established a Clean Diesel Emerging Technologies Program to help companies purchase innovative technologies to control fleet-related diesel emissions. Technologies existed. The aim was to make innovative, targeted technologies available and financially accessible to companies.

In funding based on the pull model, research may be influenced by decision makers, either by focusing on priorities, emergencies, threats, or an urgent need for data. For example, an infectious outbreak requires rapid knowledge and treatment solution, which requires that research priorities become oriented by decision makers. Programs that targeted science included the development of influenza vaccines (the NIVAREC center funded by ZonMw), the development of specific, applied knowledge (*e.g.*, ZonMw’s Chronic Fatigue Syndrome Program, which encompasses the science of chronic fatigue syndrome, treatment and rehabilitation methods), programs based on priority areas such as Aboriginal research (SSHERC), and strategic awards (NHMRC).

The funding agencies that we studied all funded either push, pull, or L&E research (Table 3, line 1). Many agencies funded initiatives based on at least two models (push and pull), but these projects represented less than one-third of all documented funding (all models).

### Types of internal actions

Internal activities carried out by the agencies themselves help develop organizational knowledge and SIPP. The internal activities that we documented varied in nature. Some activities reinforced capabilities, such as classes and SIPP training sessions, or helped build organizational knowledge (capability). Some activities were more dynamic and helpful in building organizational knowledge, such as creating knowledge brokers, forums, and communities of practice. They put actors into situations where they could discuss and/or solve problems or become more comfortable with specific topics or issues; in short, they helped build organizational knowledge understood as a process. Other activities favored the provision of operationalized information through guides and handbooks. These helped the organization build knowledge understood as a codification. Capability, process, and codification are ways to categorize knowledge [28], and an organization can use any of these as a basis of its actions.

### Activities that reinforce capabilities

Most funding agencies offered services that facilitated the use of evidence-based knowledge in decision making. Some resources, like online portals, assisted in identifying and tracking funding opportunities (NHMRC). Three-day courses or a center for posting reviews allowed researchers to learn and share experiences (NIHR) and diffuse success stories, to share ideas and good practices with other grant applicants and practitioners (ARC, NSERC, CIHR and INSERM). Publishing lists of licensed innovations created opportunities for companies to carry out discovery research (INSERM). And classes, summer schools (CIHR), and lecture sessions for researchers, policy makers, or parliament members (CIHR, AR) served to train actors for SIPP.

### Activities that reinforce processes

Our documentation shows that not only resources, but also active mechanisms favored SIPP. These mechanisms put the utilization of science in the decision-making process in a dynamic context that enabled researchers and decision makers to interact through networks of trainees (CIHR), encouraged journalistic interest in science discoveries through journalism awards (CIHR), enabled companies to consult groups of research expertise (INSERM), and allowed professionals to interact through communities of practices (NHMRC). EPA had policy agreements that allowed for formal liaisoning and the communication of scientific data to decision makers and official regional coordinators for research and development.

### Activities that reinforce codification

Making formalized knowledge useable relies on informative and descriptive documents. Summaries of research results can be translated into useful tools for practitioners, like the targeted summary of guidelines by NHMRC. Among informative documents, we also found knowledge transfer handbooks (CIHR). To translate research findings, the funding agencies also developed more prescriptive tools, such as targeted summaries of guidelines (NHMRC) or specific guidelines (NIH, USA, EPA, USA), and even rules (EPA, USA), agreements, and policies (NIHR).

In summary, we found that the agencies and ministries developed a variety of activities, especially capabilities, to make knowledge useful and useable (Table 3, line 3). These activities enriched the organizations and enabled them to use the science and knowledge produced.

### Steps of innovation

Funding for eligible expenses through funded programs, which may be based on a push, pull, or L&E model, may cover a number of different steps in the innovation cycle [12]. The first step in innovation is initiation, in which connections between decision makers and researchers are established and topics of interest fine-tuned. The second step is development, in which the research is conducted and then data collected and analyzed. Then there is synthesis (end of grant), which involves the presentation of research results to the public through conferences and written diffusion methods like reports. Sometimes reports synthesize previous results in the field. Research efforts may center on either generic research topics, priority areas, or SIPP itself. An eligible applicant, such as a fellowship candidate, institution or group of actors, may, for example, benefit from classes on SIPP or even do a temporary stay at a federal institution in order to get a direct feel of how and what is done inside public administration (CIHR). The goal of such training is to help the applicant translate research into polity and practices.

The most common steps that were funded in the agencies we studied were end of grant, research development, and training in SIPP (Table 3, line 2). Only two agencies funded all steps in the innovation cycle. While development and end-of-grant projects appeared to be more frequently funded than knowledge synthesis or SIPP training projects, the funding agencies did sometimes have internal SIPP-related activities that were not related to funding competitions.

We may draw the following conclusions about the funding agencies’ involvement in the innovation process:

1. The push, pull and L&E models of knowledge transfer were each supported by a slight majority of funding agencies

2. Few of the funding agencies relied on all three models, meaning that all of the complementary dynamics of the knowledge processes were not always covered in their funding efforts;

3. Externally funded programs largely covered SIPP at the end-of-grant stage of the innovation process, promoting diffusion in reviews, conferences, etcetera;

4. Internally developed SIPP activities mainly focused on providing technical support, such as lectures via an online portal, and such activities were less frequent than those aimed at bridging and translating knowledge.

### Analysis of benefits

Tracking health service research utilization is not an easy task [3,29]. Here, we report on SIPP-related success stories, providing examples, individual cases, and positive results in which funded activities have led to changes in the use of resources for service delivery, changes in the production of service delivery, or in policy making.

### Types of benefits

The funding agencies mentioned some benefits for practice and policy of their SIPP-related support. We did not find any references to the impact of SIPP-related internal activities. The granting agencies reported the consequences of SIPP under headings like ‘impact spotlights,’ ‘impact stories,’ ‘outcomes,’ and ‘results.’ The benefits discussed in published reports took two forms: direct results of the funded research, such as a decrease in the level of cholesterol in the intervention group, and the impact of the funded research beyond the immediate scientific community, such as an influence on decision making in the form of informing guidelines used to develop a new policy. NHMRC’s Evaluation and Outcomes Working Committee defines direct results as the knowledge creation outcome of research [30].

For the purposes of this research, we are interested in the indirect outcomes and influences of SIPP benefits. These benefits encompass both macro and micro issues. They emerge at the policy level, at the practice/production level, and at the level of service delivery to clients and the population (Table 3, line 4).

### Benefits at the policy level

The modifications of policies by SIPP funding could have local, national, or international effects (Table 3, line 4). When the modifications were local, they affected the practices of groups or organizations. Sometimes national policies were modified through adjustments to regional or federal policies. The third type of SIPP influence was international, whereby the policies of international institutions or non-governmental organizations made changes to their policies based on practices elsewhere in the world.

### Benefits at the practice level

Some of the SIPP funds supported operational modifications aimed at improving processes and procedures. These mechanisms were sometimes implemented at the country level (Table 3, line 4). When regulations changed under the impetus of research data, the result could be the launch of a brand new process or the alteration of existing ones. Alterations could be accomplished through the deletion of outdated information that requires modifications, or though the addition of information to improve current practices. The modification or introduction of a new practice or regulation on practices should, theoretically, lead to improved service delivery. For engineering projects that lead to conclusive outcomes, technologies could be used by other industries, thereby allowing for the creation of new products, diagnostic tools or production lines.

### Benefits at the service delivery level

Eventually, research outcomes served as a basis for the adjustment of services delivered to the population—to patients, to clients, and to other countries (Table 3, line 4). Service delivery requires human resources, material resources and financial investment. We documented adjustment in all of these types of resources based on evidence gathered in research.

We traced the reported success stories to a number of different fields: policy making, practice organization, and service delivery (Table 3, line 4). A common impact of the funding agencies funds was the adjustment of practical rather than policy aspects, and science delivery. The funding agencies, especially those focused on medical development and technology, often reported the commercialization of research ideas—for example, the development of vaccines and medical technologies. While success stories related to SIPP were often reported under sections pertaining to the immediate results of research, the influence of research on service delivery, practice, and policy making may not be consistently gathered through funded projects nor reported in funding agencies like CNRS.

To summarize, our analysis of funding agencies revealed the following:

1. There is evidence of benefits at the policy, practice and service delivery levels;

2. There is evidence that CIHR publicly documents SIPP benefits in a more consistent way than do the other funding agencies studied;

3. There is evidence that the benefits of SIPP on practice are more common than reported.

## Discussion and conclusions

### Conclusions

The goal of this paper was to address the following questions: what visibility do funding agencies give to SIPP? How do funding agencies promote SIPP? What impacts do funding agencies have on practice and policy? It is difficult to draw a complete and accurate portrait of SIPP due to the diversity of terms being used to designate knowledge transfer and evidence-informed decision making for policy and practice. This no doubt reflects the fact that this is still a field under construction. Our findings support those of Tetroe *et al.*[25], who found that funding agencies from a variety of countries defined ‘knowledge transfer’ in many different ways [25]. Their results were based on interviews with health research funding agencies, ministries, non-governmental organizations, researchers, and on an analysis of the funding and internal activities of the agencies.

Apart from the variety of models, definitions, and measures that agencies use, there is a need to clarify individual and organizational brokering interventions [31]; reduce inconsistency in publication dissemination measurement and guidance [32]; and adapt health research systems to policy makers and to stakeholders’ policy-making systems [5,33]. We need to take into consideration issues related to researcher autonomy (knowledge, career based on productivity, employment stability); policy motivation (agendas, urgent needs, career based on publicity, re-election); and the closeness of funding agencies to researchers and policy maker*s*. Based on our results, we would also add such factors as scarcity of activities or funds to cover SIPP, from the early stages all the way up to the final steps of innovation.

In our study, the importance attributed to SIPP was revealed through an analysis of how present it was in the structure of the funding agencies. Some of the agencies defined knowledge translation in their mission statement and corporate objectives, while others did not. Some agencies created a specific branch, directorate or department to address SIPP.

The funds available to cover SIPP were embedded in push, pull, and linkage and exchange models. Few of the funding agencies that we studied offered funding based on all three of these models. Comparing, between these three models, how the results of a funded project on a particular topic is translated into knowledge is worth investigating further. Indeed, using a combination of all three models may lead to more significant research utilization than does favoring one model over another.

While funding agencies emphasized the importance of SIPP, much work still needs to be done to ensure coverage of all models of the knowledge transfer process and of all steps in the SIPP process. This lack is seen in our analysis of the programs being funded and in their internal activities. The benefits obtained from funding SIPP and from SIPP activities are difficult to measure and compare because data reporting and collection is generally not systematic, as others have also noted [5]. Practice integration is largely predominant over policy integration, which is in line with Kastrinos’ point that there is ‘a marked trend away from mission-oriented policies and towards diffusion-oriented policies’ [19]. Funding agencies are becoming more responsive to science than they are to policy [34].

### Study limitations

While some of the funding agencies studied showed a broad range of internal activities aimed at promoting SIPP, the published documentation of internal activities, either through websites or official documentation, was not consistent across data sources. What is needed is more comparable data, and the integration of unpublished internal SIPP activities in development. This paper certainly does not provide an exhaustive list of the resources or mechanisms available for SIPP. Internal activities created within the agencies, or across them, were not always documented in reports and webpages accessible to the public, while pilot activities under development were not yet available. Nonetheless, we are confident that we have covered the sections considered important by the funding agencies, as such sections are generally the most clearly identified sections.

The empirical secondary data that we gathered from our sources are limited: they do not necessarily reflect the most recent and up-to-date decisions of funding agencies. We also need to consider that the benefits of research are mainly self reported by the researchers themselves, and that these benefits may be the result of either funding for the execution of research or funding aimed at encouraging the use of research by stakeholders.

Furthermore, the relationship between the models (push, pull, and L&E models), or the different steps that are funded (synthesis, initiation, development, end of grant, science of SIPP, training in SIPP), and the actual results obtained have yet to be investigated. Additionally, the success stories purporting to demonstrate the actual integration of science into decision making seemed to depict the consequences of research results on practice, and less on service delivery or even policy. Thus, our analysis probably under represents the extent of internal activities related to SIPP and underestimates the results of funding. However, a quality verification across websites and some internal documents reporting on the activities of two Canadian institutions, Health Canada and CIHR, yielded the same general results.

### Contributions

This paper makes an important contribution to research on SIPP because it goes beyond the two community model, which is based on the conceptualization that researchers produce knowledge and end users utilize knowledge, to include a variety of actors other than just producers and users of science. Other authors have pointed to the importance of having researchers frame the policy program [7], and to the major roles played by funding, research and policy actors. We focus on such issues in a detailed way, examining how science is integrated into policy and practice by an important actor in this process—funding agencies.

One needs to keep in mind that while in this paper we have conceptualized producers as researchers from the scientific community, it is not always easy to make a distinction between producers and users of science. For example, some producers of scientific knowledge work inside national departments and agencies. Similarly, users of knowledge for decision making can be found in a host of different entities: health organizations like hospitals; civil servants assigned to decision making positions within funding agencies, civil servants outside the Ministry of Health, such as the Ministry of the Environment; politicians deciding on health-related legislation; even university researchers whose work builds on previous research. This heterogeneous sample of users may require a host of user-specific mechanisms for the initiation, development and dissemination of research.

This paper sets the foundation for further discussion on SIPP in funding agencies in the healthcare domain. We have detailed the complex relationships that exist between knowledge use in the process of decision making at both the policy and practice levels. The other element—in essence, the flip side—worth investigating is knowledge development. This aspect, the integration of policy and practice into science, may be essential if we are to achieve a meaningful dialogue and reciprocal influence between research and policy, and between research and practice [7]. Indeed, the production of new and useable knowledge to improve policy and practice might find interesting lessons in the study of how policy and practice integrate into science. Insights for the integration of policy into science can be learned from previous studies on the Rothschild initiative in England [16,17]. Researchers conducted a seven-year formative evaluation of follow-up data on this attempt by the English Department of Health in the 1970s to influence and set the agenda for healthcare research. Recent editorials on collaborative research have highlighted the importance of maintaining an active relationship between research and the policy and practice side [11].

Case studies are very useful for identifying best practices in evidence-informed decision making. Of course, the integration of knowledge/science into practice and policy does not depend on funding agencies alone [35]. Other organizations like, for example, Canada’s Policy Research Institute, inform policy, as do government entities like deputy ministers. In countries like Germany, there are mechanisms for such integration on advisory boards and government task forces [5]. Future research could focus on whether establishing specific priority areas for funding is more effective in informing decision making than is funding based on pull, push, or L&E models.

## Competing interests

This study was funded in part by Health Canada. This funding agency did not participate in the writing of this paper; nor did it read or approve it.

## Authors’ contributions

PS conceptualized and designed the study, and then collected and analyzed the data. Both PS and JLD interpreted the data. PS wrote the initial version of the paper, which was then revised by both PS and JLD. Both authors read and approved the final manuscript.

## Supplementary Material

Additional file 1 List of documentation analyzed. Click here for file
