# Supplementary material for: How research funding agencies support science integration into policy and practice: An international overview
Source: Implement Sci. 2014 Feb 24;9:28. doi: 10.1186/1748-5908-9-28 (PMC3939639; doi:10.1186/1748-5908-9-28)
Supplement: Additional file 1 — List of documentation analyzed. [file 1748-5908-9-28-S1.doc]

### Additional file 1. List of documentation analyzed

| Agence Nationale de la Recherche. Web site. NA [cited 2011 March-  April]; Available from: <http://www.agence-nationale-recherche.fr/>.  Australian Research Council. Strategic Plan 2010-11 to 2012-13. 2010.  Australian Research Council. Web site. NA [cited 2011 March-April]; Available from: [www.arc.gov.au/](http://www.arc.gov.au/).  Canadian Institute of Health Research. 10 to your health Celebrating 10 years of research success. CIHR-IRSC; 2010.  Canadian Institute of Health Research. Knowledge Translation 10 year Report 2010.  Canadian Institute of Health Research. Impacts of CIHR funded research: a compendium of results Communications and public outreach. . CIHR-IRSC; 2010.  Canadian Institute of Health Research. Knowledge to action 2009-2010 CIHR-Supported Health Research at Work for Canada and Canadians. NA.  Canadian Institute of Health Research. Departmental Performance Report 2009-2010. In: Canada TBo, editor. NA.  Canadian Institute of Health Research. Report on Plans and Priorities 2009-2010. In: Canada TBo, editor. NA.  Canadian Institute of Health Research. Web site. CIHR-IRSC; NA.  Conseil d'administration du Centre National de la Recherche Scientifique. "Horizon 2020" plan stratégique du CNRS. 2008 [cited 2011 March]; Available from: <http://www.cnrs.fr/fr/organisme/docs/Plan_Strategique_CNRS_CA_080701.pdf>.  Environmental Protection Agency. Office of science policy website. NA; Available from: <http://www.epa.gov/osp/>.  Environmental Protection Agency. Web site. NA [cited 2011 March-April]; Available from: <http://www.epa.gov/>.  Inserm Transfert mission. Web site. NA [cited 2011 March]; Available from: [www.inserm-transfert.fr/](http://www.inserm-transfert.fr/).  National Institutes of Health. Web site. NA [cited 2011 March]; Available from: <http://www.nih.gov/about/mission.htm>.  National Health and Medical Research Council. Testing the NHMRC’s urgent research process. Strategic research development committee of the national health and medical research council, SRDC occasional paper number 1. 2000.  National Health and Medical Research Council. 10 of the best NHMRC funded health & medical research successes. 2006.  National Health and Medical Research Council. Strategic plan 2010-2012. 2010.  National Health and Medical Research Council. Web site. NA; Available from: <http://www.nhmrc.gov.au/>.  National Institute for Health Research. The National Institute for Health Research Version 3 (July 2011). 2011.  Natural Sciences and Engineering Research Council (NSERC). Departmental Performance Report 2009-2010. In: Canada TBo, editor. NA.  Natural Sciences and Engineering Research Council (NSERC). Report on Plans and Priorities 2009-2010. In: Canada TBo, editor. NA.  Natural Sciences and Engineering Research Council (NSERC). Impact stories web site pages. NA; Available from: <http://www.nserc-crsng.gc.ca/Media-Media/ImpactStories-ArticlesPercutants_eng.asp>.  Natural Sciences and Engineering Research Council (NSERC). Web site NA [cited 2011 March-April]; Available from: [http://www.nserc-crsng.gc.ca](http://www.nserc-crsng.gc.ca/).  Research Centre for Science and Technology studies. Science policy. Universiteit Leiden,; 2012 [cited 2012 April].  Social Sciences and Humanities Research Council of Canada (SSHRC). Departmental Performance Report 2009-2010. In: Canada TBo, editor. NA.  Social Sciences and Humanities Research Council of Canada (SSHRC). Report on Plans and Priorities 2009-2010. In: Canada TBo, editor. NA.  Social Sciences and Humanities Research Council of Canada (SSHRC). Web site. NA [cited 2011 March-April]; Available from: [www.sshrc-crsh.gc.ca](http://www.sshrc-crsh.gc.ca/).  The Netherlands Organisation for Health Research and Development. ZonMw corporate brochure. NA; Available from: <http://www.zonmw.nl/fileadmin/cm/admin/documenten/ZonMw_corporate_EN_site.pdf>.  The Netherlands Organisation for Health Research and Development. Web site. NA [cited 2011 March-April]; Available from: [http://www.zonmw.nl](http://www.zonmw.nl/). |
| --- |
